# CATCH-ON Educational Interventions for Providers, Older Adults, and Caregivers

**DOI:** 10.1093/geroni/igab046.1909

**Published:** 2021-12-17

**Authors:** Erin Emery-Tiburcio, Michelle Newman, Robyn Golden

**Affiliations:** 1 Rush University, Chicago, Illinois, United States; 2 Rush University Medical Center, Chicago, Illinois, United States

## Abstract

CATCH-ON, the collaborative GWEP led by Rush University Medical Center, is working to create Age-Friendly Communities by assuring that health systems, community-based organizations, and older adults and families are educated about the 4Ms. For providers, CATCH-ON offers a monthly Learning Community that focuses on one of the 4Ms each quarter. Each session provides practical recommendations for 4Ms implementation and opportunities to share experiences in small groups. CATCH-ON also partnered with Community Catalyst, older adults, and caregivers to develop a 4Ms educational brochure. The brochure is available electronically and by paper to educate older adults and caregivers about the 4Ms and discussing them with their healthcare team. Additionally, CATCH-ON created 4M online modules for older adults and families. This session will explore the success and lessons learned in developing educational interventions for diverse audiences and how this approach strengthens Age-Friendly Communities.

